# Colchicine treatment can be discontinued in a selected group of pediatric FMF patients

**DOI:** 10.1186/s12969-022-00780-w

**Published:** 2023-01-04

**Authors:** Keren Cohen, Shiri Spielman, Rotem Semo-Oz, Guy Bitansky, Maya Gerstein, Yonatan Yacobi, Asaf Vivante, Irit Tirosh

**Affiliations:** 1 Sackler Faculty of Medicine, Tel-Aviv University, Tel-Aviv, Israel; 2 Paediatric Rheumatology unit, Edmond and Lily Safra Children’s Hospital, Sheba Medical Centrer, 52621 Ramat-Gan, Tel Hashomer Israel; 3 Department of Paediatrics A, Edmond and Lily Safra Children’s Hospital, Sheba Medical Centre, Tel Hashomer, Israel; 4 Department of Paediatrics B, Edmond and Lily Safra Children’s Hospital, Sheba Medical Centre, Tel Hashomer, Israel

**Keywords:** Colchicine, FMF, *MEFV* gene, Paediatrics, Treatment

## Abstract

**Objectives:**

Familial Mediterranean Fever (FMF) patients are required to adhere to a life-long treatment with colchicine, primarily for preventing amyloidosis. As some patients may be asymptomatic for long periods of time, it remains unclear whether it is possible to discontinue colchicine treatment in a selective group of patients. We aimed to identify predictive characteristics for a successful cessation of colchicine therapy.

**Methods:**

Out of 646 FMF pediatric patients followed in our referral FMF clinic, colchicine treatment was discontinued in 51 patients. In this study we compared the genetic, demographic, and clinical characteristics between patients for whom a successful cessation of therapy was made (Group 1; *n* = 21) and patients for whom cessation of therapy was deemed a failure (Group 2; *n* = 30) and consequently had to resume colchicine therapy.

**Results:**

Patients for whom a successful cessation of therapy was achieved had no biallelic pathogenic *MEFV* mutations, were less likely to have “severe attacks” (two or more FMF characteristic symptoms) (24% vs 80%; *P* = 0.000067) and did not require higher than 1 mg/day of colchicine, prior to the drug cessation. Remission duration under colchicine treatment was significantly higher in group 1 compared with group 2 (4.36 years ±2.12 vs 2.53 years ±2; *P* = 0.0036).

**Conclusion:**

This study supports the concept of colchicine free remission in a minority of FMF patients (3%). Holding treatment, under close monitoring, may be reasonable when selecting the appropriate patients.

## Background

Familial Mediterranean Fever (FMF) is an autoinflammatory disease, characterized by recurrent, self-limited attacks of fever and serositis [1] and when untreated can lead to Serum Amyloid A (SAA) amyloidosis [2]. The mainstay of FMF treatment is colchicine, which reduces the frequency as well as the intensity of disease exacerbations. Colchicine also alleviates the inflammatory response and prevents progression to amyloidosis [3].

The diagnosis of FMF is based mainly on clinical criteria and most diagnostic criteria do not require the presence of *MEFV* mutation for final diagnosis. Nonetheless, genetic analysis is routinely performed. Although there is no one definite diagnostic test for FMF, once diagnosis is made, patients are treated with a life-long prophylactic colchicine, mainly in order to prevent amyloidosis [4]. While colchicine is relatively well tolerated, it can cause adverse reactions and toxicity. Moreover, patients may experience a life-long treatment as a burden.

As some patients may be asymptomatic for long periods of time, the question of whether it is possible to discontinue colchicine treatment - in a selective group of patients - arises. In recent years, few studies showed colchicine-free remission in FMF patients [5–8] but there are no detailed recommendations to whom colchicine cessation can be tried.

In this study, we reviewed 51 medical records of children diagnosed with FMF, for whom treatment with colchicine was discontinued following a long period of remission. We compared genetic, demographic, and clinical characteristics between two groups: patients for whom a successful cessation of therapy was made and patients who had to resume colchicine therapy.

The aim of this study was to identify patient’s characteristics that can predict a successful cessation of colchicine therapy in pediatric FMF patients.

## Methods

### Study participants

This retrospective study was conducted at the pediatric section of the Israeli National Center for FMF at Sheba Medical Center between 2007 to 2020. Of 646 medical records of patients diagnosed with FMF based on Tel-Hashomer criteria and the Eurofever/PRINTO criteria, colchicine treatment was discontinued in 51 patients following a disease remission (no FMF clinical attacks and normal inflammatory markers) of a minimum of 1 year (mean of 3.28 years). After treatment cessation, follow up was recommended every 4-6 months. During follow-up, treatment was re-initiated for patients who experienced one of the following: (1) recurrence of symptoms; (2) elevated acute phase reactants (ESR or CRP); (3) elevated SAA.

Patients were grouped as follows: Group 1, included patients who remained clinically and laboratory asymptomatic without colchicine (*n* = 21). Group 2 included patients who required reinitiating of colchicine (*n* = 30).

Clinical and laboratory data were documented for both groups including clinical manifestations such as episodes of fever, arthritis, abdominal pain, chest pain, erysipelas like erythema and scrotal pain, inflammatory markers such as ESR, CRP and SAA, demographic data, relevant family history, and genetic analysis of *MEFV* mutations. The extent of MEFV gene testing was altered during the study period. As of 2010, all patients underwent exon 10 sequencing. Before 2010, several specific mutations were tested which included at least three predominant mutations (p.M694V, p.V726A and p.E148Q) covering 88% of the mutations identified in the Israeli FMF population.

Approval for human subjects’ research was obtained from the Institutional Review Board of Sheba Medical Center, approval number 6148-19-SMC.

### Disease severity score

Disease severity was calculated according to Pras severity criteria [9]. Pras Score reflects the perspective of the disease course and includes the maximal colchicine dose used; maximal frequency of attacks; and an overview of the entire clinical manifestations of the disease throughout the follow-up period and therefore was calculated at the patients’ last appointments.

### Statistical analysis

Results are given as proportions or mean ± standard deviation as appropriate. Differences between the groups in discrete variables were evaluated by Chi-square test. Comparisons of continuous variables were done by unpaired Student t-test in a 2-sided analysis; *p* value < .05 was considered significant.

Time-to-event analysis was performed using Kaplan-Meier plots and the log rank test to assess significance. Cox regression analysis was used to determine study covariates and time to relapse of FMF events using *p* value of 0.05 for inclusion; hazard ratio (HR) 95% confidence interval (CI) was calculated.

Statistical analysis was performed using R software (version 3.6.3, R Foundation for Statistical Computing, Vienna, Austria).

## Results

### Colchicine cessation

**Fig. 1:**
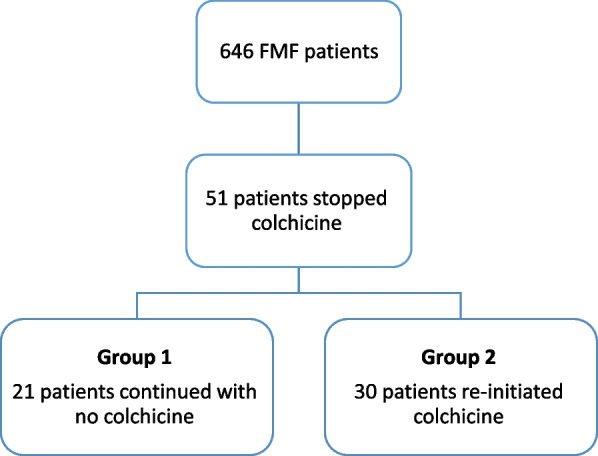
Patients Flowchart

During the study period, 51 patients (7.9%) had a trial of colchicine cessation. Specifically, in 47 of these patients, colchicine was discontinued by their primary rheumatologist while in the other four, it was stopped per their parents’ decision. As seen in Fig. 1. discontinuation of colchicine was successful for 21 patients (group 1), while 30 patients required re-initiation of colchicine (group 2). Colchicine was restarted due to recurrence of clinical symptoms (21 patients) or elevated SAA (9 patients); mean SAA in group 2 was 18.6 mg/l. All patients in group 1 had normal inflammatory markers during follow up: mean ESR 8.9 mmHg, mean CRP 0.9 mg/l and mean SAA 3.2 mg/l.

### Patients’ characteristics

Family history of FMF was higher (*p* = 0.0327) in group 2 (Table 1). Furthermore, remission duration under colchicine treatment was significantly longer in group 1 compared with group 2 (4.36y ± 2.12 vs. 2.53y ± 2; *p* = 0.0036). There was no significant difference between the two groups regarding other demographic characteristics.

**Table 1:** Demographic and clinical characteristics of study groups

Characteristics	Group 1 *N* = 21	Group 2 *N* = 30	*p* value
**Male/female (n)**	10/11	17/13	0.5241
**Age at disease onset**	4.14 ± 3.08	4.42 ± 3.74	0.7737
**Family history of FMF (%)**	48	77	**0.0327**
**Duration of colchicine treatment, years**	6.35 ± 2.76	5.69 ± 4.33	0.9958
**Remission duration under colchicine treatment**	4.36 ± 2.12	2.53 ± 2	**0.0036**
**Follow up duration off colchicine treatment, years**	4.52 ± 2.96	3.44 ± 3.27	0.2295
**FMF attacks (%)**			
Fever	95	97	0.7959
Peritonitis	76	90	0.1820
Arthritis	33	70	**0.0096**
Pleuritis	5	20	0.1196
ELE	0	3	0.3981
Orchitis	0	10	0.1352
**Severe attacks** ^a^**(%)**	24	80	**0.000067**
**Non-attack manifestations (%)**			
Exertional leg-pain	10	50	**0.0025**
**Treatment (%)**			
Colchicine dose > 1 mg/day	0	40	**0.0009**
**Pras Score**			
Pras SCORE	7.14 ± 1.56	8.43 ± 1.57	**0.0058**
Pras SCORE ≥ 9 (%)	19	43	0.0701

^a^Severe attacks are characterized by fever and two or more of the following: peritonitis, arthritis, pleuritis, erysipelas like erythema and orchitis

Detailed clinical characteristics of FMF related symptoms are seen in Table 1. Patients who experienced two or more of clinical symptoms were defined as having “severe attacks” and they were more common in group 2 (80% vs. 24%; *p* = 0.000067). Furthermore, musculoskeletal symptoms including arthritis and exertional leg pain were significantly more prevalent in group 2 (33% vs. 70%; *P* = 0.0096 and 10% vs. 50%; *p* = 0.0025, respectively) and the calculated Pras score was significantly higher in group 2 (8.43 ± 1.57 vs. 7.14 ± 1.56; *p* = 0.0058).

### Colchicine dose

All patients in group 1, who remained colchicine free, required a colchicine dose of no more than 1 mg/day prior to the drug cessation while 40% of patients in group 2 required colchicine at higher doses (0% vs 40%; *P* = 0.0009).

### Relapse probability

**Fig. 2:**
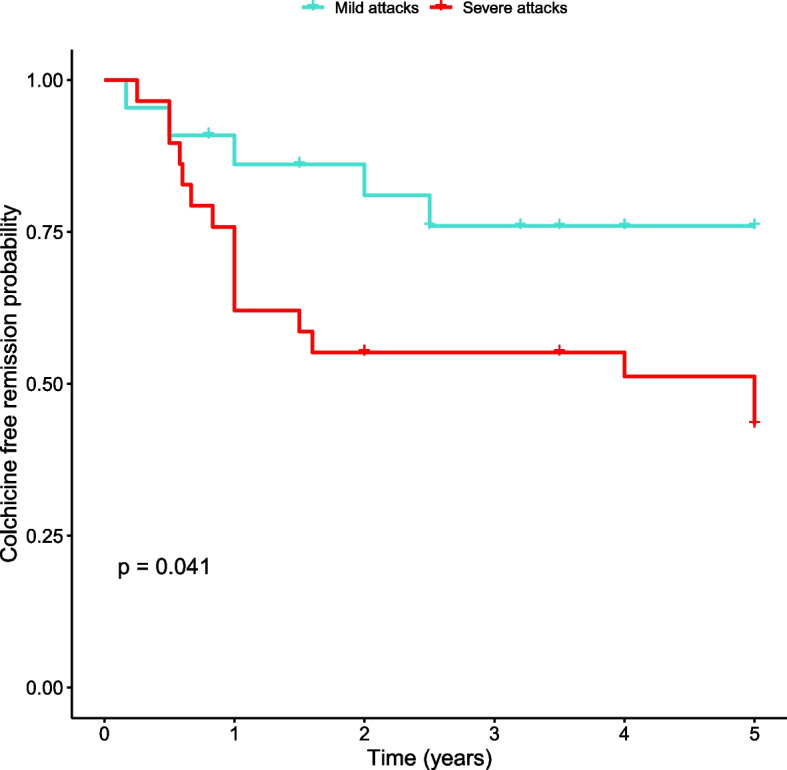
Time to re-initiating colchicine in patients with severe attacks*. Kaplan-Meier analysis of the cumulative probability of relapse among patients with severe attacks (blue) compared to patients with mild attacks (red). *Severe attacks are characterized by fever and two or more of the following: peritonitis, arthritis, pleuritis, erysipelas like erythema and orchitis

**Fig. 3:**
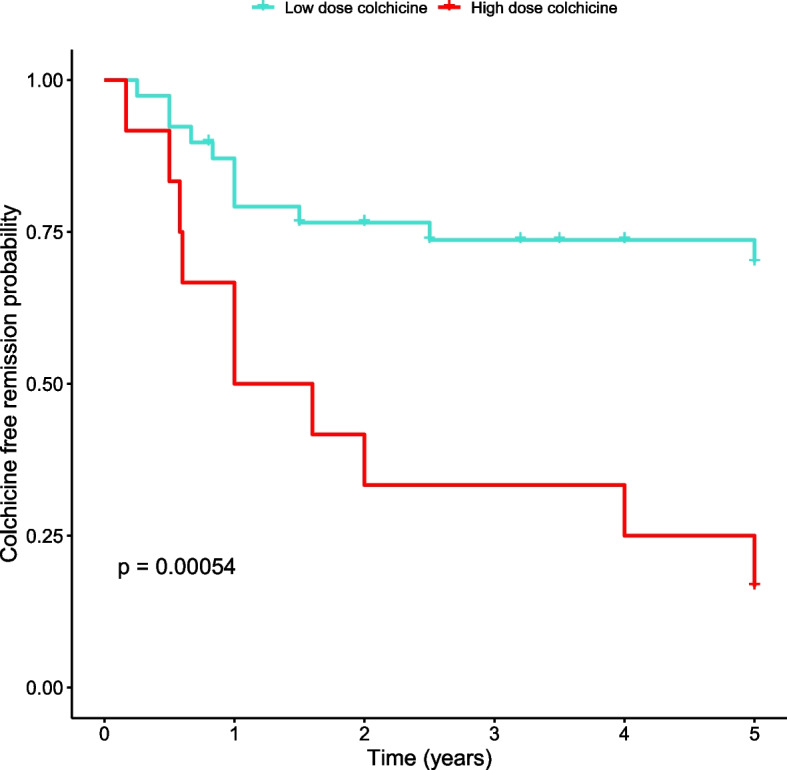
Time to re-initiating colchicine in patients with high colchicine dose*. Kaplan-Meier analysis of the cumulative probability of re-initiation of colchicine among patients requiring low (red) vs. high (blue) colchicine dose. *High colchicine > 1 mg/day

We observed a higher cumulative probability of relapse during the follow up period among patients with “severe attacks” compared to patients with mild attacks (log-rank *p* = 0.041) demonstrating a Hazard Ratio (HR) of 3.23 (95% CI = 1.004 to 7.49, *p* = 0.0492) (Fig. 2). Similarly, a higher cumulative probability of relapse was noted in patients treated with colchicine dosage of > 1 mg per day prior to drug discontinuation (log-rank *p* = 0.00054) (Fig. 3) yielding a HR of 4.09 (95% CI = 1.73 to 9.69; *p* = 0.0013).

### Genetic analysis

We observed that none of the patients in group 1 harbored biallelic pathogenic *MEFV* mutations. Table 2 shows *MEFV* mutations in the study groups. None of the patients were homozygous to *MEFV* p.M694V mutation while the number of patients with compound heterozygosity (harboring two of the following mutations: p.M694V, p.V726A or p.A744S) were exclusively noted in group 2 (0% vs. 33%; *P* = 0.0032). Four patients had no known pathogenic mutations, one in group 1 and three in group 2 (5% vs. 10%; *P* = 0.4935). Overall, only two patients had missing genetic information (5% vs. 3%; *P* = 0.7959).

**Table 2:** *MEFV* mutations in the study groups. n (%)

Genotype	Group 1 *N* = 21	Group 2 *N* = 30	total *N* = 51	*P* value
**M694V/0**	14 (67)	9 (30)	23 (45)	**0.0096**
**M694V/V726A**	0 (0)	9 (30)	9 (18)	**0.0057**
**M694V/A744S**	0 (0)	1 (3)	1 (2)	0.3981
Two pathogenic variants (Total)	0 (0)	10 (33)	10 (20)	**0.0032**
**M694V/E148Q**^a^	3 (14)	6 (20)	9 (18)	0.5983
**V726A/E148Q**^a^	2 (10)	1 (3)	3 (6)	0.3551
**No mutations**	1 (5)	3 (10)	4 (8)	0.4935
**N/A**	1 (5)	1 (3)	2 (4)	0.7959

^a^E148Q should not be considered as a pathogenic variant [10]

## Discussion

FMF is viewed as a chronic disease requiring a lifelong treatment, although colchicine-free remission periods were previously described in certain FMF patients [5]. The 2016 European League Against Rheumatism (EULAR) treatment recommendations for FMF [11] suggested colchicine dose reduction in patients with no attacks for more than 5 years as well as lack of elevated acute phase reactants. Subsequently, in 2017, Sönmez et al. were the first to report active discontinuation of colchicine treatment in asymptomatic FMF patients harboring heterozygous *MEFV* variants [7]. Similarly, in 2019 Tanatar et.al [6]. showed that in 2.6% out of a cohort of 1786 FMF pediatric patients, the authors were able to safely discontinue colchicine treatment. These patients remained in remission for median of 3.1 years (37.4 months) during the study follow-up.

Our study further supports this clinical approach demonstrating that there are certain FMF patients in whom colchicine may be discontinued. In the current study, we discontinued colchicine treatment in 51 out of 646 FMF patients followed in our center. Twenty-one patients were able to stay off colchicine treatment for a mean follow up of more than 4 years. Thus, a long-term successful discontinuation of colchicine was achieved in 3.2% of subjects in our cohort, in line with previous reports (Sonmez-2.8%, Ben-Zvi-3.3%).

It should be noted that successful and durable remission off colchicine is clinically relevant only for carefully selected group of patients. For the vast majority of FMF patients, colchicine continues to be a highly potent life-long treatment.

Our study provides further data to the evolving concept of colchicine cessation. Specifically, we report clinical outcomes of a longer duration of colchicine-free follow-up (mean 4.52 years / median 58 months) and a detailed description regarding the clinical presentation and genetics of patients with successful discontinuation of colchicine. This analysis has therefore prompt us to propose clinical scenarios in which when co-exist, cessation of colchicine under a tight follow-up, may be considered (Table 3). The recommended scenarios in Table 3 are consistent with clinical data reported in previous studies, particularly the genetic categorization. None of the patient in our study or the previous mentioned studied had two pathogenic mutations. Tanatar et al. [6] showed that shorter time on colchicine lead to a higher risk of relapse which support the second criteria in Table 3. In Ben-Zvi’s study, patients in the remission group had milder severity of FMF and responded well to a lower dose of colchicine in correspondence with our third and fourth criteria. Our recommendations add to Butbul et al. [8]; who suggested considering discontinuation of colchicine in patients with attack-free period of at least 6 months, absence of 2 mutations in the MEFV gene, normal levels of acute-phase reactants, no family history of amyloidosis, and no proteinuria.

**Table 3:** Clinical scenarios that may trigger consideration for colchicine cessation trial when co-exist

1. Patients without biallelic *MEFV* pathogenic mutations (^a^).	
2. Patients with at least 4 years of remission under colchicine treatment.	
3. Patients who required daily colchicine dose of 1 mg or less to maintain remission.	
4. Absence of history of severe attacks (^b^).	

^a^E148Q should not be considered as a pathogenic variant [10]

^b^Severe Attacks = fever and two or more of the following: peritonitis, arthritis, pleuritis, erysipelas like erythema and orchitis

It is important to emphasize that a close clinical and laboratory follow up is required following treatment cessation. We recommend following those patients every 4 months with a careful assessment for any clinical symptom of FMF (such as unexplained fever, serositis, ELE), elevated inflammatory markers (ESR, CRP, SAA) or proteinuria. If all signs and symptoms are negative, then continuing with a close follow up off-colchicine is reasonable. Importantly, in group 2 we re-initiate colchicine safely following close monitoring due to clinical or laboratory features of active disease. All responded well and none had proteinuria.

There are several limitations to our study. This is a retrospective study and therefore the data is limited and the number of patient is small. However, our data includes the longest follow up of a large pediatric cohort reported to date. A second limitation is the possibility that few patients were misdiagnosed with FMF. Having said that, all patients were diagnosed by an experienced pediatric rheumatologist and fulfilled the diagnostic criteria and therefore should be considered as having FMF.

## Conclusions

This study supports the concept of colchicine free remission (remission while not taking colchicine) in a minority of FMF patients (3%). Holding treatment may be reasonable when selecting the appropriate patients and under close monitoring. As current data on colchicine cessation are limited and retrospective, a prospectively designed trial is required to test the hypothesis that alleviating the lifelong colchicine burden in FMF patients is feasible.

## Data Availability

The datasets used in the current study are available from the corresponding author on reasonable request.

## Abbreviations

FMF: Familial Mediterranean Fever
SAA: Serum Amyloid A
EULAR: European League Against Rheumatism

## References

[1] Livneh A (1997). Criteria for the diagnosis of familial Mediterranean fever. Arthritis Rheum.

[2] Gafni J, Ravid M, Sohar E (1968). The role of amyloidosis in familial mediterranean fever. A population study. Isr J Med Sci.

[3] Ben-Chetrit E, Levy M (1998). Colchicine: 1998 update. Semin Arthritis Rheum.

[4] Zemer D (1986). Colchicine in the prevention and treatment of the amyloidosis of familial Mediterranean fever. N Engl J Med.

[5] Ben-Zvi I (2014). Colchicine-free remission in familial Mediterranean fever: featuring a unique subset of the disease-a case control study. Orphanet J Rare Dis.

[6] Tanatar A (2019). Short-term follow-up results of children with familial Mediterranean fever after cessation of colchicine: is it possible to quit?. Rheumatology (Oxford).

[7] Sonmez HE (2017). Discontinuing colchicine in symptomatic carriers for MEFV (Mediterranean FeVer) variants. Clin Rheumatol.

[8] Aviel YB (2021). Discontinuation of colchicine therapy in children with familial Mediterranean fever. J Rheumatol.

[9] Pras E (1998). Clinical differences between north African and Iraqi Jews with familial Mediterranean fever. Am J Med Genet.

[10] Tirosh I (2021). Clinical significance of E148Q heterozygous variant in paediatric familial Mediterranean fever. Rheumatology (Oxford).

[11] Ozen S (2016). EULAR recommendations for the management of familial Mediterranean fever. Ann Rheum Dis.

